# Molecular microevolution and epigenetic patterns of the long non-coding gene *H19* show its potential function in pig domestication and breed divergence

**DOI:** 10.1186/s12862-016-0657-5

**Published:** 2016-04-23

**Authors:** Cencen Li, Xiao Wang, Huimin Cai, Yuhua Fu, Yu Luan, Wen Wang, Hui Xiang, Changchun Li

**Affiliations:** Key Lab of Agriculture Animal Genetics, Breeding, and Reproduction of Ministry of Education, College of Animal Science and Technology, Huazhong Agricultural University, Wuhan, 430070 People’s Republic of China; State Key Laboratory of Genetic Resources and Evolution, Kunming Institute of Zoology, Chinese Academy of Sciences, 32 East Jiaochang Road, Kunming, Yunnan Province 65022 China; Graduate School of the Chinese Academy of Sciences, Beijing, 100049 China; Department of Computer Science, City University of Hong Kong, Hong Kong, 999077 China; BGI Co Ltd, Shenzhen, 518083 China; South China Normal University, Guangzhou, 510631 China

**Keywords:** Pig, Long non-coding gene, *H19*, Molecular evolution, Methylation, Domestication, Breed divergence

## Abstract

**Background:**

The domestic pig *Sus scrofa domesticus* originated from the wild boar *S. scrofa* about 10,000 years ago. During domestication, drastic morphological, physiological, and behavioral changes developed between domestic pigs and wild boars through artificial and natural selection. The long non-coding RNA (lncRNA) *H19*, which is located within the imprinting gene cluster *H19-IGF2*, plays an important role in regulating muscle development in humans and mice. This study systematically analyzed the molecular evolution of *H19* and its possible epigenetic changes during pig domestication and breeding to explore the genetic and epigenetic contributions of *H19* to pig domestication.

**Results:**

The molecular evolution of *H19* was initially analyzed on a large phylogenetic scale. Results showed that the gene was highly conserved within a broad range, especially in the 5′ terminal sequence. The molecular evolution of the gene was then analyzed using published re-sequencing data of 30 wild boars from Tibet, 3 wild boars from Sichuan, and 15 native pigs from other regions in China. Eight polymorphic sites were identified, and the nucleotide diversity (π) value within the *H19* gene body was significantly higher (Z-test, *P* < 0.05) in domesticated pigs than in wild pigs. However, no significant divergence occurred between domesticated and wild pigs. Single nucleotide polymorphisms in the 3′ terminal sequence were surveyed in other Chinese local breeds and foreign pig breeds. We observed a consistently higher diversity in domesticated pigs than in wild pigs. The methylation pattern of the *H19* gene in pigs was subsequently analyzed using published methylated DNA immunoprecipitation data and an unpublished single-base resolution liver methylome. Analysis results showed distinct methylation levels in some tissues. Among the samples surveyed, Landrace showed the lowest methylation level, followed by the Guizhou wild boar, whereas the Enshi pig exhibited the highest methylation level in the 2 kb upstream region of the *H19* gene. Liver transcriptome data suggested that Landrace harbored the highest expression of the *H19* gene, followed by the Guizhou wild boar, whereas the Enshi pig harbored the lowest expression of the gene. Differential methylation sites (DMSs) among the three breeds were mainly identified in the 2 kb upstream region of the *H19* gene. In the Enshi pig, we detected allele-specific methylation (ASM) regions in the 2 kb upstream region of the *H19* gene. Most of the DMSs in the upstream 2 kb region of the gene were also located in the ASM region in this breed.

**Conclusions:**

Molecular analyses suggest that the *H19* gene was highly conserved during large-scale evolution and exhibited genotype differentiation during domestication and breed differentiation. The drastic diversity pattern between domestic and wild pigs in the *H19* gene body, which was highly conserved during large-scale evolution, suggests that this gene might have played roles in the breed differentiation of domestic pigs. Methylation analysis indicates an opposite epigenetic regulation direction between Chinese and European pig (EU) domestication, which resulted in opposite expression changes in this gene between the two domesticated groups. Our preliminary analyses on DMSs among different pig breeds and ASM imply that imprinting was associated with methylation differences. This study systematically demonstrates the genetic and epigenetic patterns of *H19* during pig domestication and provide valuable cues and basis for further research on the function of *H19* in pig domestication.

**Electronic supplementary material:**

The online version of this article (doi:10.1186/s12862-016-0657-5) contains supplementary material, which is available to authorized users.

## Background

Pigs are important domesticated animals that provide an important source of meat in China and the whole world. Pigs in East Asia originated from the East Asian wild boar and were domesticated approximately 10,000 years ago [1]. Natural and artificial selection have promoted the development of significant differences in morphology, growth, fertility, and local fitness between domesticated and wild pigs [2]. Various Chinese local pig breeds have been produced through domestication and breeding. Statistical data from the United Nations Food and Agricultural Organization (FAO) indicated that 118 native pig breeds, which accounted for 24 % of the global count, were available in China in 2000 [3]; these breeds may be classified into six types: North China (NC), South China (SC), Central China (CC), Jianghai (JH), Southwest China (SW), and Plateau. Diverse breeds of local pigs present valuable genetic resources [4]. Pigs from different regions show substantial variations in physiological features. For instance, Taihu pigs from JH exhibit high fecundity; CC- and SC-type pigs show high adipose contents, which possibly contribute to the good taste of meat from these pigs; and Plateau- and NC-type pigs contain more lean meat than fat. Clearly, pigs from different regions in China have developed different characteristics that have enabled them to adapt to particular environments and conditions during microevolution. However, the molecular evolutionary mechanisms underlying the diversity of the same traits among different pig breeds during domestication and improvement remain unclear. Several genes plays important roles in species domestication and breed divergence [5–8]. As key factors in pig domestication, non-coding RNAs have been speculated to serve important functions in pig domestication and breeding [9].

Non-coding genes are DNA sequences that produce RNA with no translational ability [10]. Most non-coding RNAs can regulate gene expression [9]. The long non-coding gene *H19* is located in the *H19/IGF2* imprinted gene cluster. Studies on mice and human have shown that the *H19* gene contributes to the growth, development, and generation of muscle fibers [11–14].

Aside from genetic mutations, epigenetic mechanisms also influence gene expression and explain how gene-environment interactions yield particular phenotypes during development [15]. In particular, some complex genetic phenomena cannot be explained by DNA variations alone, but integrated with epigenetic systems, such as DNA methylation with strong reversibility, microRNA regulation or histone modification, which can be passed to subsequent generations [16]. Recent studies have suggested that the *H19* gene influences embryonic growth and development as well as skeletal muscle differentiation and regeneration via epigenetic mechanisms, such as DNA methylation. Abnormal methylation of the *H19* gene may be associated with cancer formation [17, 18]. In addition, research on the epigenetic mechanisms underlying Beckwith-Wiedemann Syndrome (BWS) [19] and Silver-Russell Syndrome (SRS) [20] has shown that methylation of the *H19* gene exerts a pronounced effect on disease and muscle development. Pidsley [21] analyzed DNA methylation and genotypes in three differential methylated regions (DMRs) and investigated the *H19* promoter in the *H19/IGF2* cluster in humans; results showed that DNA methylation is strongly associated with cerebellum weight [21]. These findings indicate that *H19* methylation exerts a pronounced effect on disease and muscle development.

Pigs are highly domesticated animals that drastically vary in meat features depending on the breed and geographic location. The *H19* gene contributes to the skeletal muscle growth and development of animals through both genetic variation and epigenetic modification. Therefore, *H19* was speculated to play a vital role in porcine domestication and breed divergence via genetic mutation and/or epigenetic changes.

To understand the molecular evolution pattern and methylation pattern of the *H19* gene in relation to pig domestication and breed divergence, the *H19* gene and its flanking region were analyzed using the homologous sequence of 18 species by Phast software [22] and Mega software [23]. Results revealed that the *H19* gene is relatively conservative in a large-scale evolution. Published re-sequencing data [24] of 30 wild boars from Tibet, 3 wild boars from Sichuan, and 15 native pig breeds from other regions in China were initially used to detect single nucleotide polymorphisms (SNPs) in the *H19* gene. Most SNP sites were distributed in the 3′-terminal of the *H19* gene. Candidate SNPs from 14 Chinese native pig breeds and 3 foreign pig breeds (a total of 234 individuals) were also subjected to clone sequencing to survey the SNPs obtained using polymerase chain reaction (PCR). The nucleotide diversity and F‑statistics (F_st_) along the upstream, gene body, and downstream regions of the *H19* gene were analyzed using the re-sequencing data of 30 Tibetan wild boars, 3 wild boars from Sichuan, and 15 Chinese native pig breeds. The *H19* gene was conserved during domestication and had a high divergence in the domestication pig population, indicating the functional role of this gene. Separate methylated DNA immunoprecipitation sequencing (MeDIP-Seq) data were obtained from eight adipose tissues and two muscle tissues of three specimens of Tibetan wild boars, Rongchang pigs, and Landrace pigs. Single base-resolution bisulfate methylation sequencing data of liver tissue from one Chinese Enshi pig, one Landrace pig, and one Guizhou wild boar were also obtained (unpublished data). Data from MeDIP-Seq and bisulfite sequencing were then used to compare the methylation patterns of the *H19* gene between domesticated pigs and boars. The possible role of the *H19* gene during pig domestication and breed divergence was systematically described on the basis of molecular evolution analysis results, epigenetics differences, and gene expression data.

## Results

### Evolution of *H19* among mammals

**Fig. 1:**
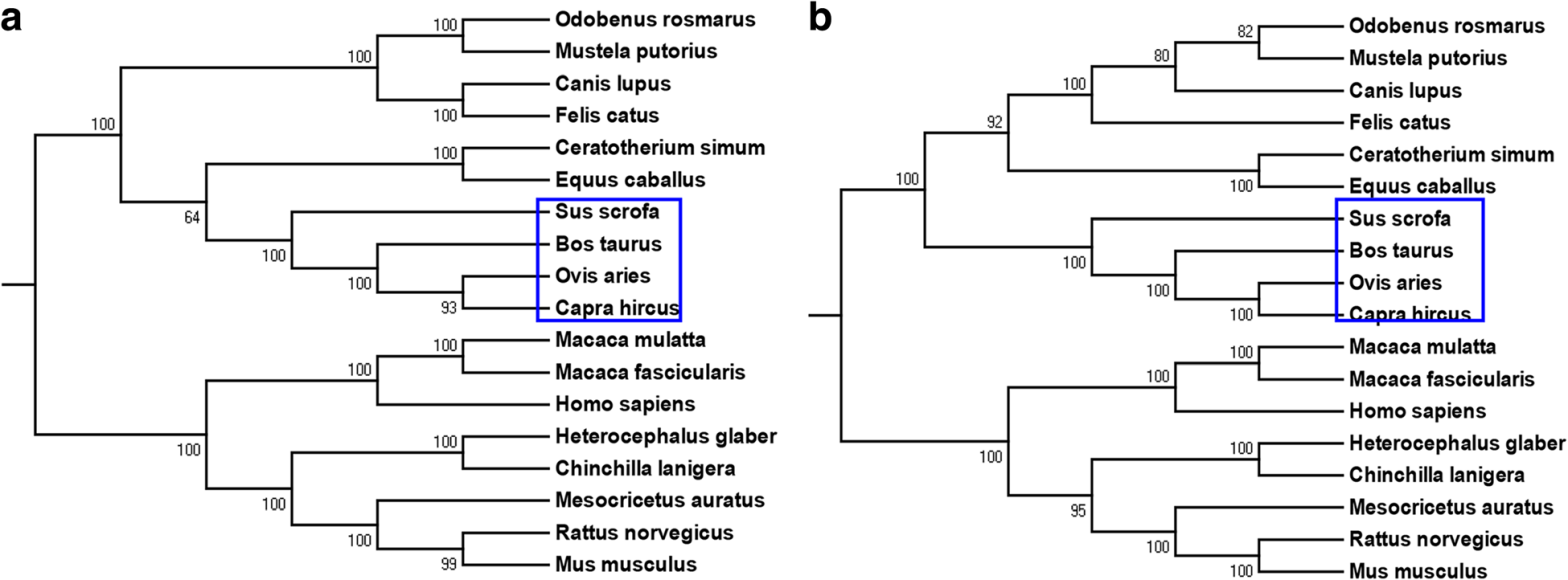
Molecular Phylogenetic analysis by Maximum Likelihood method. The evolutionary history of *H19* gene (**a**) and mitochondria of 18 species (**b**) were inferred by using the Maximum Likelihood method based on the Kimura 2-parameter model [43]. The bootstrap consensus tree inferred from 1000 replicates [43, 44, 54, 55] is taken to represent the evolutionary history of the taxa analyzed. Branches corresponding to partitions reproduced in less than 50 % bootstrap replicates are collapsed. The percentage of replicate trees in which the associated taxa clustered together in the bootstrap test (1000 replicates) are shown next to the branches

The evolution of the *H19* gene was first analyzed on a large phylogenetic scale to assess its evolutionary status. Basing from the *H19* gene transcript sequence and mitochondrial sequence of 18 mammals, we constructed a gene tree and a phylogenetic tree by using MEGA6. The gene tree (Fig. 1a) was largely consistent with the phylogenetic tree (Fig. 1b). Results showed that primates and rodents cluster into independent clades and exhibited a close relationship than *Sus scrofa*.

**Fig. 2:**
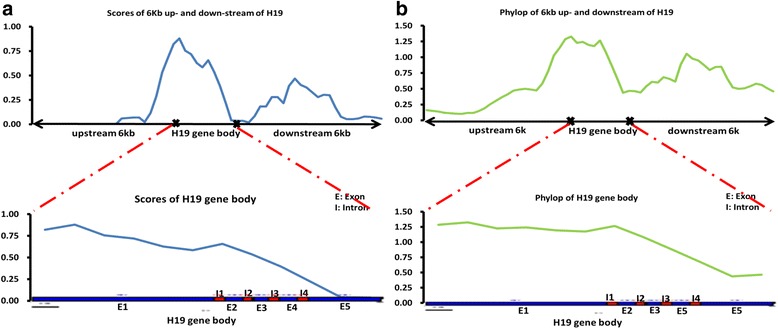
PhastCon score and phyloP value of *H19* among 18 species [22]. The phastCons score (**a**) and the phylop value (**b**) of *H19* gene body among 18 species was calculated and then was calculated using sliding window for segments of 1000 bp with 300 bp intervals

Conservation scores were calculated using PhastCons [22] to prove that the *H19* gene was conserved during large-scale evolution and to identify the conserved and accelerated evolutionary regions in the gene among the 18 species. Results showed that the upstream region of the *H19* gene was lowly conserved whereas the gene body, specifically exon1, was highly conserved (Fig. 2a, Additional file 1: Figures S1A and S2A). PhyloP was used to calculate the *p*-value of conservation or acceleration, and results indicated that the *H19* gene was conserved during evolution within a broad range (Fig. 2b, Additional file 1: Figures S1B and S2B). The evolution of *H19* on a large phylogenetic scale indicates that the gene was highly conserved within abroad range, including the 3' region of *H19*.

### Screening for selective signal

**Fig. 3:**
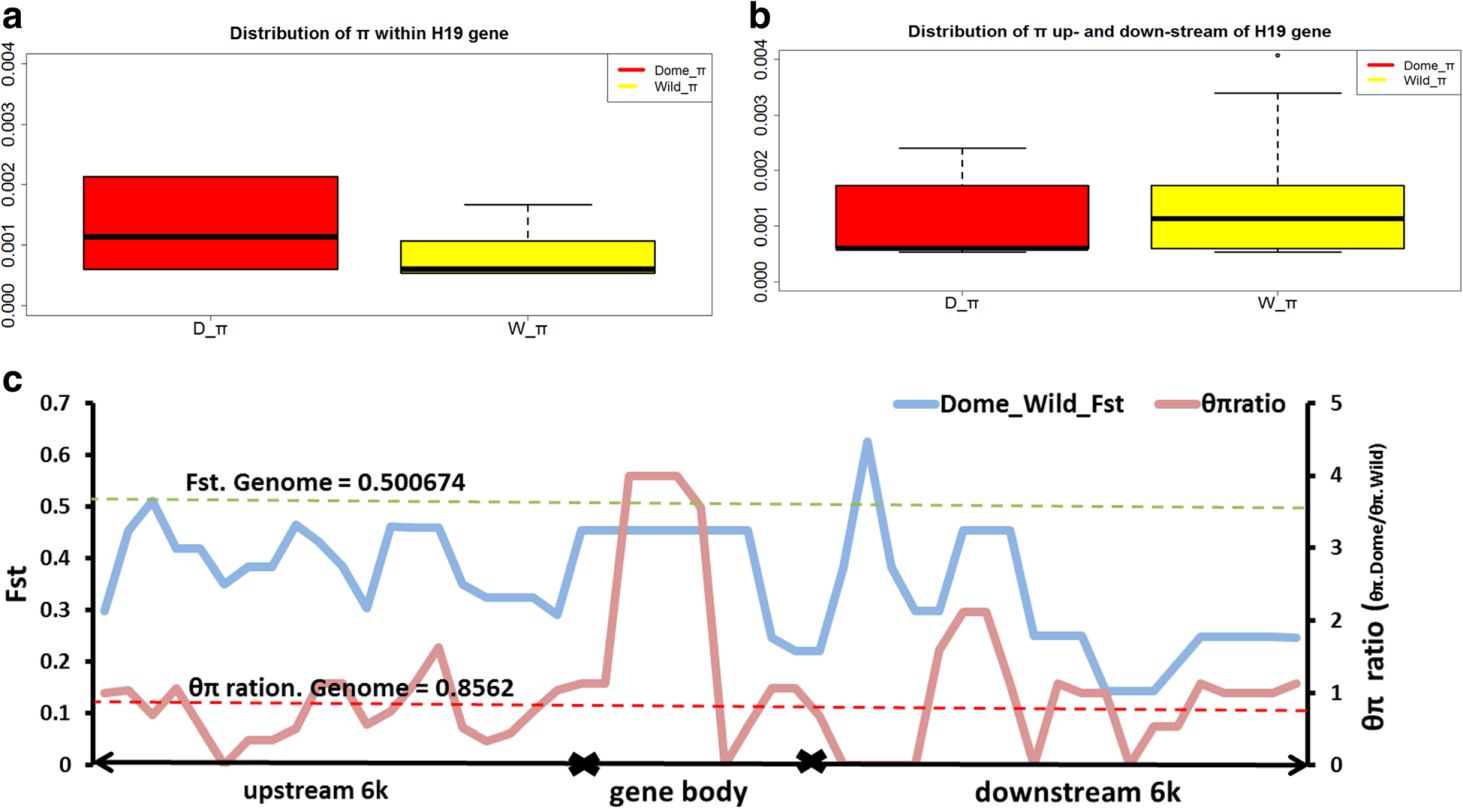
Patterns of nucleotide diversity and pair-wise population differentiation of *H19* locus in domesticated pigs and wild boars. Sliding window analysis of nucleotide diversity and genetic differentiation coefficient between groups at *H19* for domesticated pigs (Dome) and wild boars (Wild). π (right) and F_st_ (left) was calculated for segments of 1000 bp with 300 bp intervals (**c**). The distribution of nucleotide diversity (π) and F_st_ within *H19* gene (**a**) and linked upstream and downstream 6 kb (**b**) was shown in the boxplot, the middle thick black line represent median.θ_π_ ratio was calculated by π_.dome_/π_.wild_, the red dash line and green dash line respectively represent the average θ_π_ ratio and the average Fst of the whole genome of *Susscrofa*

We analyzed the published re-sequencing data [24] on *H19* to detect a selective sweep of the *H19* gene during domestication on a genomic scale. We identified eight alleles among the domestic pigs, wild boars, and Tibetan boars. Although all of the eight alleles were detected in the domestic pigs, a large portion of these alleles were homozygous in pig individuals (Additional file 1: Tables S2 and S3). This high degree of homozygosity implies that the gene is associated with pig breeding [25]. We further calculated the *π* and pairwise F_st_ of the linked upstream, gene body, and downstream genomic regions of the *H19* gene in 30 Tibetan wild boars, 15 domesticated pigs, and 3 wild boars from Sichuan with 1 kb sliding windows and 300 bp steps (Fig. 3 and Additional file 1: Figure S3). The π value within the *H19* gene body was significantly higher in domestic pigs than in wild boars (Fig. 3a and c). This result suggests that the *H19* gene sequence conservation within wild boars is sharply contrasted with the sequence diversity among the *H19* alleles found within domestic pigs. Within the domesticated pig population, the upstream and downstream regions of the *H19* gene showed a low diversity, which is similar to that in wild populations (Fig. 3b). By contrast, the *H19* gene body exhibited a high diversity (Student’s *t*-test, *P* < < 0.05, Fig. 3a). This phenomenon may be caused by selection relaxation. However, considering the abundant occurrence of breed differentiation within domesticated pigs, we suspected that *H19* might have undergone genetic differentiation during breed differentiation and pig domestication. We also detected that the F_st_ of the *H19* gene body between domesticated pigs and wild boars was higher than that of *H19* downstream (Student’s *t*-test, *P* < < 0.05, Fig. 3c).

To detect whether or not *H19* is under selection, we obtained the whole-genome nucleotide divergence (π_dome_G_ = 0.0025, π_wild_G_ = 0.0030) of domesticated and wild pigs, as well as the population difference (Fst_d_w_G_ = 0.501) between domesticated and wild pigs, by using Tibetan re-sequencing data with GATK software [26]. Comparison of the sliding windows of π value and F_st_ between domesticated and wild pigs (Fig. 3c and Additional file 1: Figure S3), showed that the domesticated pigs have a significantly higher diversity than wild pigs in the *H19* gene (Z-test, *P* = 0.0128). This results suggests that *H19* in domestic pig breeds might have experienced a more rapid evolution than that in wild ones. The F_st_ between these two populations in *H19* (F_st.*H19*_ = 0.393) had no significant difference with the average F_st_ value of the whole genome (F_st.whole_ = 0.501) (Fig. 3a) [27, 28]. However, the drastic diversity pattern between domestic and wild pigs in the *H19* gene body, which is a highly conserved region during large-scale evolution, suggests that this gene might have played roles in the breed differentiation of domestic pigs.

### Nucleotide diversity

We obtained all 2608 bp sequences in the *H19* gene, including five exons and four introns from the published re-sequencing data of Tibetan wild boars [24], to identify the polymorphism sites in the gene. Results showed that the 3’-region of the *H19* gene has abundant polymorphisms. Partial sequences of the *H19* gene from 1660 bp to 2460 bp (the transcription start site is 1 bp) were obtained from 234 pig individuals consisting of China native pigs and three foreign breeds to detect the roles of the *H19* gene in breed differentiation. SNP detection results of the *H19* gene in different pig breeds are summarized in Table 1, Additional file 1: Tables S2, S3 and Figure S4. The SNP statistics table revealed eight SNPs in the region; of these SNPs, seven were observed only in domesticated pigs and one was present in both domesticated and wild pigs. However, clear differentiation of the genotype of this SNP site was observed. The proportion of the T allele was higher than or equal to that of the C allele in wild pigs, but nearly all T alleles were observed in the Tibetan wild boars. This result indicates that this site underwent differentiation in the wild pigs. Among the Chinese breeds, a number of populations were C-alleles homozygous, and other populations showed a particular frequency of T-alleles. At the population level, the SNPs were present in the heterozygous state, indicating that these sites have not been completely fixed. Interestingly, the SNP at 1818 bp exhibited a high mutation proportion in the CC and SC populations but was nearly completely homozygous in the other populations.

**Table 1:** SNP detection of *H19*

Position	Type	NC (*n* = 23)	CC (*n* = 42)	JH (*n* = 29)	SC (*n* = 84)	SW (*n* = 23)	EU (*n* = 33)	WB.S (*n* = 3)	TB (*n* = 27)
1664	C	C	C	C	C/T_(80/4)_	C	C	C	C
1818	G	G	G/A_(31/11)_	G	G/A_(50/34)_	G	G/A_(32/1)_	G	G
1993	C	C	C/T_(22/20)_	C	C/T_(69/15)_	C	C/T_(29/4)_	C	C
2020	C	C/T_(20/3)_	C/T_(37/5)_	C/T_(21/8)_	C	C	C	C/T_(1/2)_	C/T_(26/1)_
2028	G	G/A_(20/3)_	G	G	G/A_(78/6)_	G	G	G	G
2050	C	C/T_(19/4)_	C/T_(27/15)_	C	C/T_(45/39)_	C/T_(21/2)_	C/T_(26/7)_	C	C
2292	C	C/T_(21/2)_	C/T_(36/6)_	C/T_(18/11)_	C/T_(81/3)_	C/T_(19/4)_	C	C	C
2456	C	C	C	C/T_(26/3)_	C/T_(78/6)_	C	C	C	C

*NC* North China, *JH* Jianghai, *CC* Central China, *SC* South China, *SW* Southwest China, *EU* European, *WB.S* Wild pigs of Sichuan, *TB* Tibetan Wild boar

**Fig. 4:**
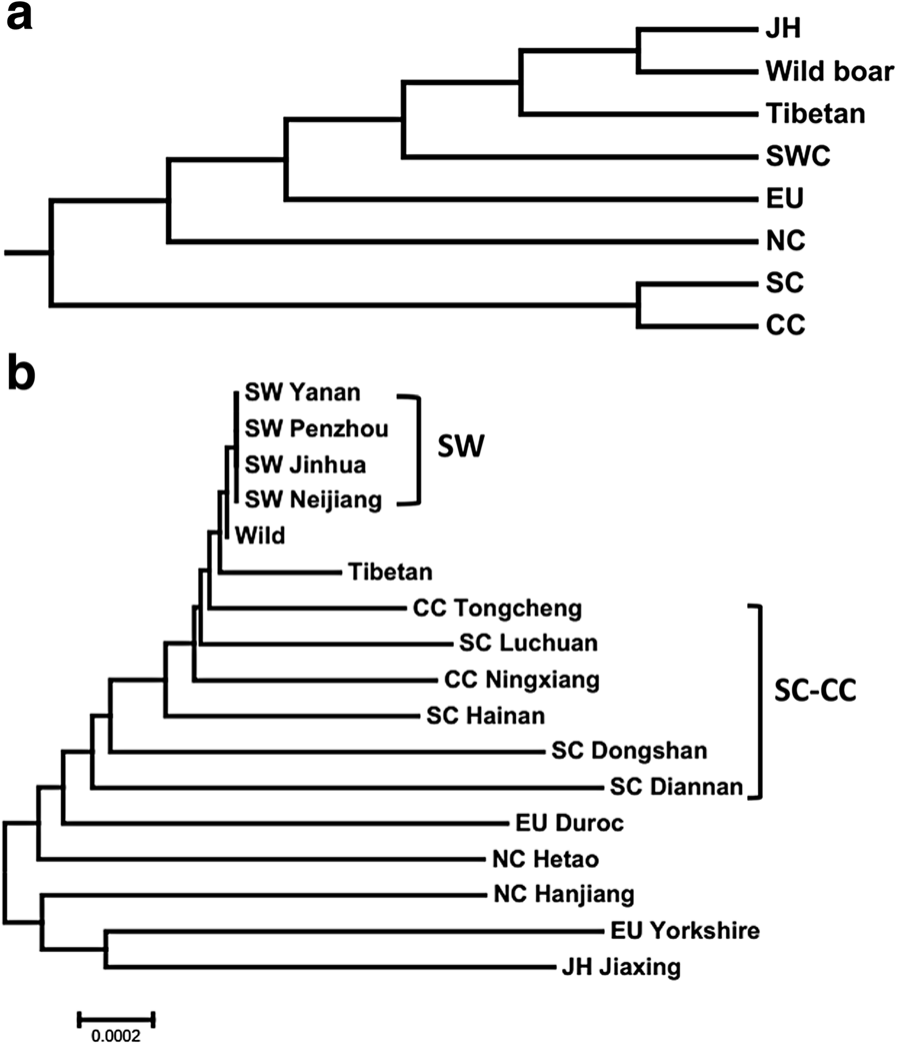
The Neighbor-joining tree of *H19* in different pig breeds. The neighbor-joining tree of *H19* base on the net distance between breeds was constructed according to catalogs (**a**) and pig breeds (**b**) by Mega 6.0

Estimates of nucleotide diversity (π) were consistently higher in pigs from CC (π = 0.00127) and SC (π = 0.00203) than in pigs from other locations, especially Tibetan wild boars (π = 0.00071) (Additional file 1: Table S4). The same findings were observed for θ and θπ (Additional file 1: Table S4). Such results suggest that genetic diversity increased during Chinese pig differentiation, although no significant selection signal was detected through Tajima’s D test [29], Fu and Li’s H and D test [30] or Fay and Wu’s H test [31] (Additional file 1: Table S4). The results of HKA test were also not significant (*P*_Dome_ = 0.8796). We constructed a neighbor-joining tree by using the net distance between populations and found that CC and SC clustered together (Fig. 4). A considerable degree of mixture possibly decreased the selection signal.

### Methylation analysis of the *H19* gene between wild and domesticated pigs

**Fig. 5:**
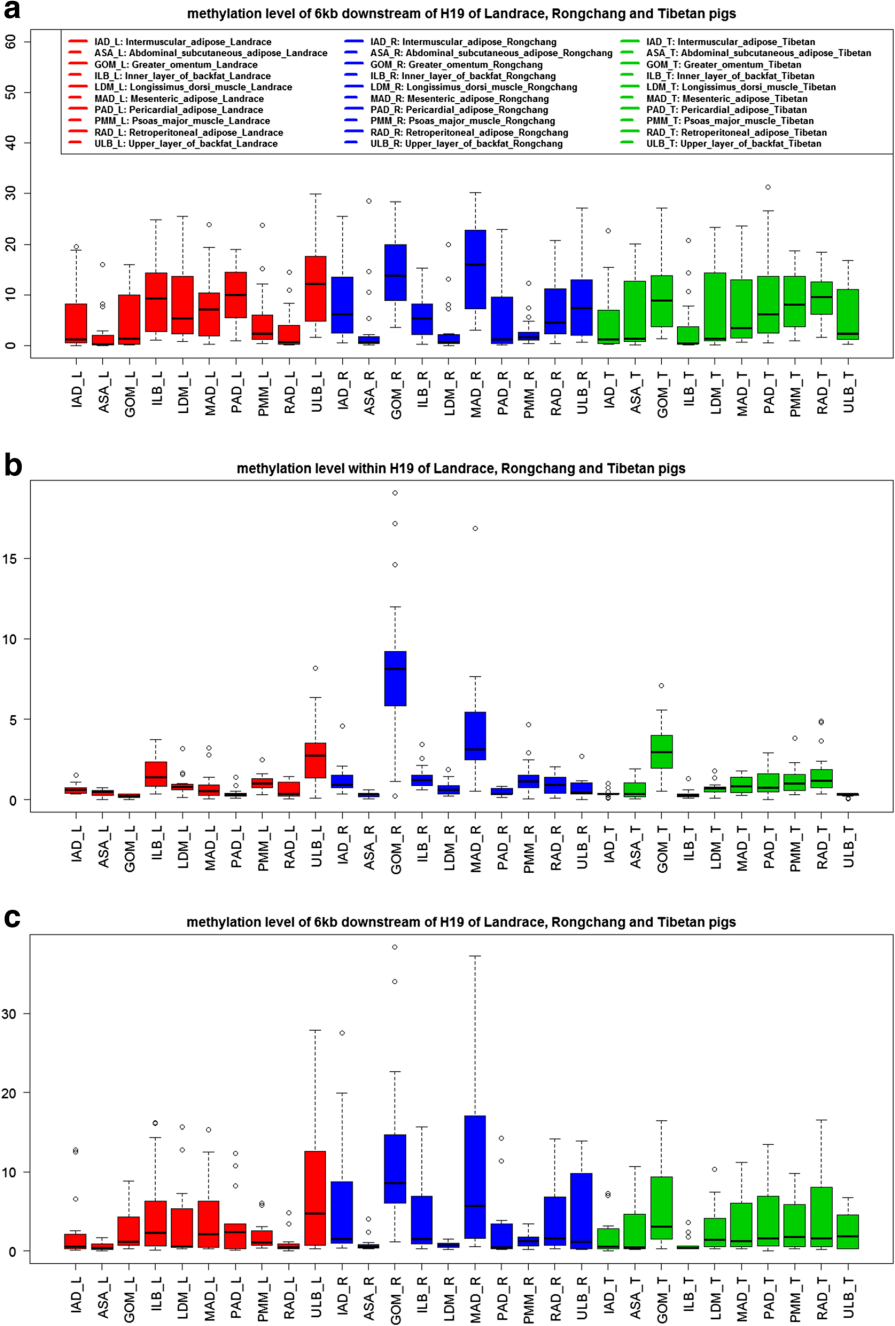
The methylation level of *H19* in 10 tissues. The methylation level of upstream 6 kb (**a**), *H19* gene body (**b**) and downstream 6 kb (**c**) of 10 tissues of Landrace (Red), Rongchang (Blue) and Tibetan (Green) pigs according to the MEDIP data. The number of reads which mapped to the reference sites represent the methylation level of sites

We selected the DNA immunoprecipitation (MeDIP) data of one tissue (longissimus dorsi muscle) of every pig sample and conducted Pearson correlation test to test whether or not a significant difference exists between the mapping results of the whole genome and the *H19* gene. Pearson correlation coefficient ranged from 0.949 to 1. This result indicates that the mapping results are highly correlated on the basis of the Pearson correlation coefficient. These results suggest that the result of whole genome mapping was consistent with that of *H19* gene mapping after validation of the selected representative data. The methylation levels of the *H19* gene body and linked upstream and downstream genomic regions were analyzed using the MeDIP data of eight fat tissues and two muscle tissues of three Tibetan pigs (the Plateau pig breed from the Tibetan wild boar lineage), Landrace (foreign pig breed), and Rongchang pig (Chinese native pig breed) to understand the methylation pattern of the *H19* gene between wild and domesticated pigs (Fig. 5 and Additional file 1: Figure S5). Results showed that the methylation levels within the *H19* gene among the three above mentioned pig breeds were lower than 6 kb upstream (Student’s *t*-test, *P* < <0.01) and 6 kb downstream (Student’s *t*-test, *P* < <0.01), whereas the methylation level was the highest in the 6 kb upstream region. Several tissues, including the greater omentum (GOM) and mesentery adipose (MAD), from the Rongchang pig showed significantly higher gene body methylation levels than the corresponding tissues in Landrace (Student’s *t*-test, *P* < <0.01) and Tibetan pigs (Student’s *t*-test, *P* < <0.01). Several tissues showed distinct levels of methylation. In Landrace, the methylation levels at both 6 kb upstream and 6 kb downstream regions in the abdominal subcutaneous adipose tissue (ASA), psoas major muscle (PMM), and retroperitoneal adipose tissue (RAD) were lower than those in other tissues, whereas the methylation levels in the upper lipid of back (ULB) were higher than those in other tissues (two-way ANOVA, *P*_tissue_ = 4.48 × 10^-6^). In the Chinese native pig (Rongchang pig), the methylation levels were obviously higher in the GOM and MAD but lower in the ASA, longissimus dorsi muscle (LDM), and PMM in all three regions (two-way ANOVA, *P*_tissue_ = 0.0308). Interestingly, the methylation levels at the upstream and downstream regions were clearly lower in the inner lipid of back (ILB) than in the gene body and other tissues of Tibetan pigs (two-way ANOVA, *P*_genebody-upanddown_ = 2.02 × 10^-12^, *P*_ILB-others_ = 1.36 × 10^-7^). The methylation patterns in ASA and PMM differed between Chinese native pigs and Tibetan pigs (Student’s *t*-test, *P* = 0.067 to 1.962 × 10^-5^) (Fig. 5).

**Fig. 6:**
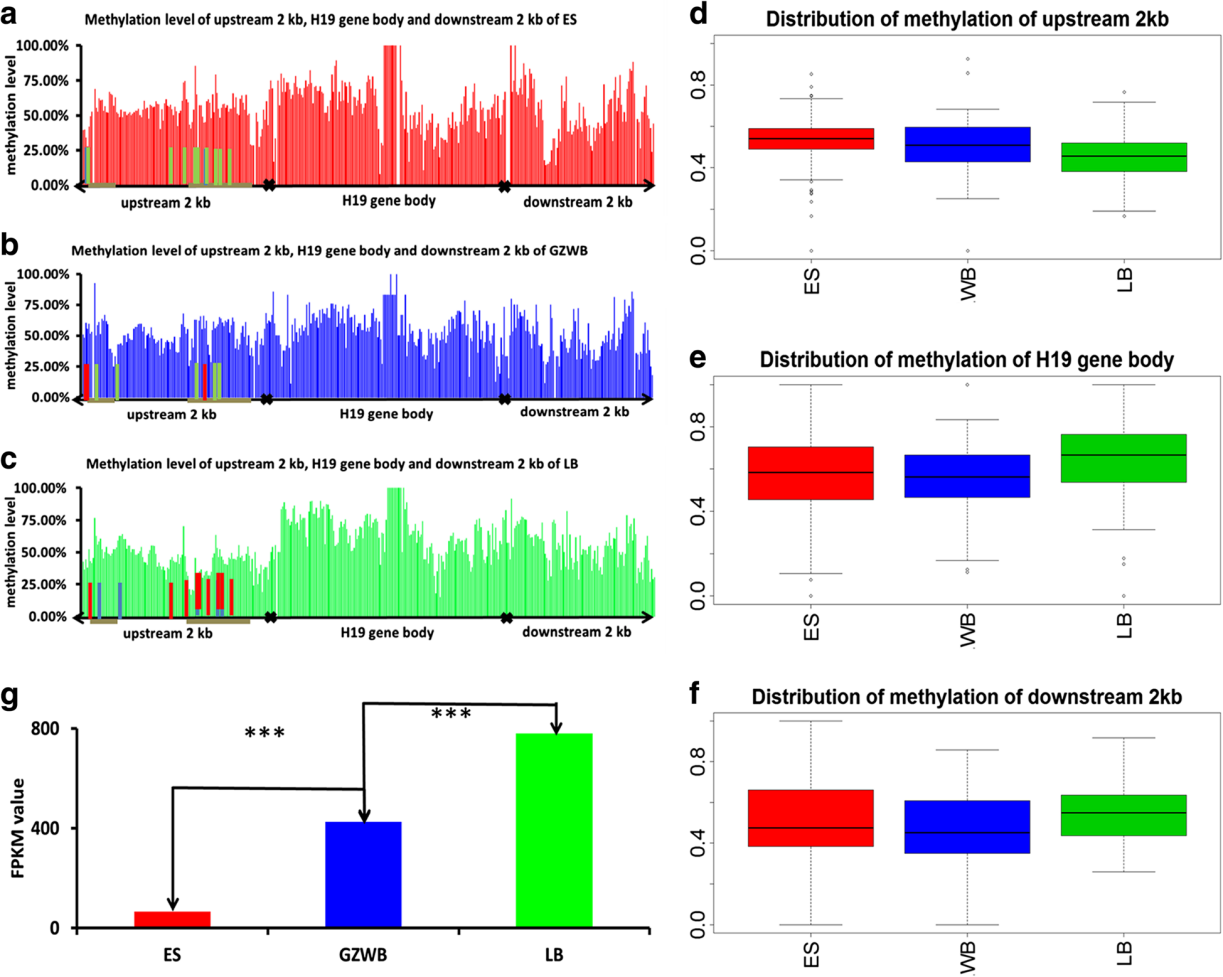
The methylation level and expression level of *H19* in liver tissue. Methylation level of upstream 2 kb (**d**), *H19* gene body (**e**) and downstream 2 kb (**f**) of ES (En Shi black pig, **a**), GZWB (Gui Zhou wild pig, **b**) and LB (Landrace, **c**) and the expression level of *H19* gene (**g**). The methylation level of every site was calculated by number of methylated reads divide number of total reads which was mapping to this site. The expression level was estimated by FPKM value which was calculated by Cuffdiff software using transcriptome data. The grey horizontal lines below the **a**, **b**, **c** were the ASM region, the vertical bars in **a**, **b**, **c** were DMS, the different color represent DMS between this pig breed and the other referred by the related color. (***P < 0.001;**P < 0.02;*P < 0.05)

The methylation levels of the *H19* gene and linked 2 kb upstream and downstream regions, as well as the expression levels in the liver, were determined and compared on the basis of our unpublished single-base liver methylome and transcriptome data of the wild boar (Guizhou wild pig), the Chinese native pig (Enshi black pig), and a foreign breed (Landrace) (Fig. 6, Additional file 2: S7). The methylation levels of the gene were the lowest in the foreign pig (Landrace), followed by that in the Chinese wild pig (Guizhou wild boar). The methylation levels in the Chinese native pig (Enshi pig) were the highest at the 2 kb upstream region of the *H19* gene (Fig. 6a–c and f). By contrast, the methylation levels in the gene body of Landrace were higher than those in the two other samples (pairwise comparisons using *t* tests, **P < 0.001 with the adjusted Bonferroni method). The expression levels of the *H19* gene were the highest in the liver of foreign pigs (Landrace), followed by those in the Chinese wild pig and Chinese native pig (Enshi pig) (one-way ANOVA, *P* < <0.01) (Fig. 6g). These results are consistent with the theory that methylation in the promoter region decreases expression levels and represses transcription noise in the gene body [32].

We further explored the relation between imprinting and methylation difference. In this step, we identified the DMSs among pig breeds and the ASM region by using our unpublished single-base resolution methylome data of the liver from the three pig breeds. MeDIP data were not in single base resolution; thus, we were unable to use the data to address tissue-specific methylated sites. We previously surveyed the methylation status of CpGs of the *H19* gene in the three breeds. In the present study, we first identified the DMSs among the three pig breeds by using Chi-square test. We found a total of 6, 9, and 12 DMSs between the Enshi pig and the Guizhou wild pig, the Guizhou wild pig and Landrace, and the Enshi pig and Landrace, respectively, in which 3, 6, and 9 sites were in the upstream 2 kb region; 2, 2, and 3 sites were in the gene body region; and 1, 1 and 0 sites were in the downstream 2 kb region, respectively. This result indicates that DMSs were mainly located in the upstream 2 kb region (Additional file 1: Table S5). We also identified regions with ASM in each breed. We identified 8 ASM regions, all of which were located in the upstream 2 kb region of the *H19* gene. Interestingly, all of these ASM regions were in the Enshi pig. In the 8 ASM regions, no SNP was found in the two other pig breeds; hence, we were unable to distinguish the allelic methylation status in these two breeds. We then used the Enshi pig as a model to explore the DMSs and ASM of *H19*. Most of the DMSs between the Enshi pig and the other pigs were located in the ASM region; specifically, 2 of 3 DMSs between the Enshi pig and the Guizhou wild pig and 7 of 9 DMSs between the Enshi pig and Landrace were situated in the ASM region. Our preliminary results imply that imprinting may be associated with methylation differences among different breeds.

## Discussion

Increasing amounts of genomic sequence data and high-throughput annotation of genes have enabled the bioinformatics analysis of various genes of interest; this analysis has consequently provided important insights into the evolutionary link between genes of interest and particular phenotypic traits associated with several diseases [33]. As one of the first long non-coding imprinting genes to be described, *H19* reportedly promotes skeletal muscle development and regeneration [14]. Epigenetic studies have reported that abnormal methylation of the *H19* gene may induce BWS syndrome [34] and influence fetal growth [35]. Thus, the *H19* gene is hypothesized to serve an important function in the evolution and breed differentiation of pigs.

To test this hypothesis, molecular evolution was first analyzed on a large scale by using the homologous *H19* sequence of 18 species. The *H19* gene was highly conserved in mammals. Relative to the upstream and downstream regions, the *H19* gene body was highly conserved, especially in the 5’-region of the gene body. The functional importance of the *H19* gene was confirmed by the relatively large-scale evolutionary perspective observed.

Molecular evolution analyses indicated a drastic divergence of diversity pattern between domestic and wild pigs. This finding is interesting because this region was highly conserved during large-scale evolution, suggesting that this region may have been under a strong evolutionary constraint. Therefore, we suspected that the obviously increased diversity within the domestic pig population might be associated with breeding differentiation. Further analyses within Chinese domestic breeds showed that several SNP genotypes within the *H19* gene clearly differentiated among the different pig breeds, with the SC and CC types showing distinct genotypes compared with the others (Additional file 1: Table S3, Fig. 4). Although we were unable to associate their genotypes with their specific meat characters, our results provide a basis for further functional efforts on the alleles we identified. Over all, the present results demonstrate that the *H19* gene serve a critical function in pig breed differentiation and local adaptation. Further functional significances on the different genotypes of this gene are needed to confirm.

*H19* is an imprinted gene. Only its maternal allele is expressed, and paternal alleles are imprinted in normal individuals. Abnormal methylation of the *H19* DMR located upstream of the *H19* transcript start site induces abnormality and diseases [36–38]. Therefore, the potential epigenetic impact of *H19* on pig domestication is an interesting issue. Maximizing both the published MEDIP data and the single-base bisulfite methylation data, we can obtain a general view of the epigenetic pattern on pigs. We found that the methylation levels within the gene body were relatively low and that tissue-specific methylation differences existed among different samples. Furthermore, we obtained a precise pattern of *H19* and successfully detected differential methylation associated with the differential expression of *H19* on the basis of our unpublished single-base resolution methylome and transcriptome data of livers from three representative pig samples. Chinese local breeds (ES) showed the highest upstream methylation but the lowest downstream methylation. Accordingly, *H19* expression in ES breeds drastically decreased compared with that in the wild boar and European breeds (LB). Promoter methylation in mammals and plants reportedly represses gene expression. The function of gene body methylation is intriguing, especially because gene body methylation may contribute to alternative gene expression [39, 40]. During Chinese pig domestication, a hyper-methylation of *H19* occurred in the liver from its wild ancestor, which affected the repression of this gene. Borensztein [13] found that Myod as a muscle source adjunction factor interacts with the *H19/IGF2* cluster to regulate myofiber differentiation and inferred that *H19* participates in growth and muscle development [13]. A recent study has also concluded that *H19* plays a crucial role in regulating skeletal muscle differentiation and generation mediated by miR-675-3p and miR-675-5p, which are encoded within *H19* by knockdown of *H19* in myoblast cells and knockout of *H19* in mouse satellite cells, respectively [14]. Thus, the inhibition of *H19* expression can influence skeletal muscle differentiation. Interestingly, epigenetic regulation on *H19* in European pig breeds is another case. Obvious de-methylation or hypo-methylation occurred in the promoter region in this breed compared with the two other breeds. Specifically, DMSs in the breed differential methylation were also identified. The methylation status of the *H19* gene in its wild ancestor, the European wild boar, remains unknown; nevertheless, the present results clearly indicated a distinctively high expression of this gene in European pig breeds and thus suggested an opposite epigenetic regulation direction between Chinese and European pig domestication. ASM plays a crucial role in epigenetic phenomenon and parental imprinting [41]. In this study, consistent to the differential methylation sites between the Enshi pig and the other breeds, all of these ASM regions were identified in the 2 kb upstream region of the *H19* gene in the Enshi pig. Our preliminary results implied that imprinting may be associated with methylation differences of *H19* genes between pig breeds. Both the differential methylation sites and ASM in the upstream region may have important impact on gene expression and further functions. Whether or not imprinting participated in epigenetic evolution during pig domestication and breed differentiation remains to be discusses in further research.

## Conclusion

The evolution patterns of the *H19* gene during pig domestication and breed differentiation were systematically analyzed at the genetic to epigenetic levels. The *H19* gene was highly conserved during large-scale evolution and exhibited genotype differentiation during domestication and breed differentiation, especially in the CC and SC populations. Epigenetic investigation showed that the *H19* gene exhibited a specific methylation pattern and suggested an opposite epigenetic regulation direction between Chinese and European pig domestication. This opposite epigenetic regulation direction resulted in opposite expression changes of this gene between the two domesticated groups. Most DMSs in the upstream 2 kb region were also located in the ASM region, which implied a great relevance between imprinting and differential methylation. Our results demonstrated the genetic and epigenetic patterns of *H19* during pig domestication and provided valuable cues and basis for further research on the function of *H19* in pig domestication.

## Methods

### Ethics

Animals care and all the experimentation in this study were carried out in accordance with the pre-approved guidelines from Regulation Proclamation No.5 of the Standing Committee of Hubei People’s Congress. All experimental procedures and sample collection methods were approved by the Institutional Animal Care and Use Committee of Huazhong Agricultural University, Wuhan, China (permit HZAUSW2015-0003). We obtained a written informed consent from the animal owners to take blood and tissue samples.

### Identification of conserved region of *H19* between 18 species

Mitochondrial DNA sequences [GenBank:NC_004029, GenBank:NC_020638, GenBank:NC_000845, GenBank:NC_002008, GenBank:NC_001808, GenBank:NC_001640, GenBank:NC_005044, GenBank:NC_006853, GenBank:NC_001941, GenBank:NC_001941, GenBank:NC_015112, GenBank:NC_021386, GenBank:NC_012920, GenBank:NC_005943, GenBank:NC_012670, GenBank:NC_013276, GenBank:NC_001700, GenBank:NC_005089), H19 homologous sequences (GenBank:XR_186971.2, GenBank:XR_001180683.1, GenBank:AY044827, GenBank:NR_027327.1, GenBank:XM_004441068.1, GenBank:NR_027326.2, GenBank:XM_013969825.1, GenBank:NR_003958.2, GenBank:AJ566210, GenBank:NR_027324.1, GenBank:XR_205565.2) GenBank:XR_260873.1, GenBank:NR_131223.1, GenBank:NR_027325.1, GenBank:XR_284039.1, GenBank:XR_001197480.1, GenBank:AF_190057.1, GenBank:XR_130973.1] and the annotation information of 18 species (Odobenus rosmarus, Mustelaputoriusfuro, Susscrofa, Canis lupus, Ceratotheriumsimum, Equuscaballus, Capra hircus, Bostaurus, Ovisaries, Rattusnorvegicus, Heterocephalusglaber, Chinchilla lanigera, Homo sapiens, Macacamulatta, Macacafascicularis, Mesocricetusauratus, Feliscatus, Mus musculus) were downloaded from NCBI. The gene tree was constructed by using the transcripts of 18 animals and the phylogenetic tree was constructed by using mitochondrial sequences of 18 animals. All the sequences was aligned by MEGA6.0 and constructed gene tree and phylogenetic tree using maximum likelihood method by MEGA6.0 respectively.

PHAST software was used to calculate the conservative score of the H19 gene among the 18 species based on the theory that mtDNA assumes neutral selection between species. Initially, phyloFit was used to fit tree models (constructed using maximum likelihood by MEGA6.0 based on the mtDNA sequences) to achieve multiple alignment of the *H19* sequences via maximum likelihood. Then, phastCons was used to generate conservation scores of the *H19* gene sequences between the 18 species listed above. Finally, phyloP was used to compute conservation or acceleration *p*-value based on the alignment of the *H19* gene sequences and a model of neutral evolution (http://compgen.bscb.cornell.edu/phast/) [22]. Results were analyzed using R studio.

### Sample collection

A list of samples used is shown in Additional file 1: Table S2. All domesticated pig breeds were collected from the local laboratory. All blood samples from domesticated pigs were collected from local farmers to represent local species. The total genomic DNA of Duroc, Landrace, and Large White pigs were extracted from tissue samples according to a standard phenol-chloroform protocol. The extracted DNA was then used to analyze the genetic diversity of *H19* of the different pig breeds.

### Cloning of the *H19* gene

Five primer pairs, shown in Additional file 1: Table S1, were designed to amplify the *H19* gene from the first exon to the last exon based on the sequence published in NCBI (GenBank: AY044827) using Primer Premier 5.0 software. Each PCR contained a final volume of 25 μL and consisted of the following components: 125 ng genomic DNA, 0.1 μM each primer, 5 μL 5 × PrimeSTAR GXL buffer (5 mM Mg^2+^ plus), 4 μL dNTP Mixture (2.5 mM each) and 0.5 U GXL Taq DNA polymerase (Takara, China). PCR conditions was set as follows: initial denaturation at 94 °C for 5 min; 38 cycles of 10 s of denaturation at 98 °C, 15 s of annealing at the appropriate temperatures listed in Additional file 1: Table S1, and 1 min extension at 72 °C; and a final 5 min extension at 68 °C. Amplified products were detected via gel electrophoresis, and the positive products were sent to BGI Tech for sequencing. All sequences were assembled using the SeqMan program in the DNASTAR software suite.

### Molecular evolution analysis

First, DNA sequences were aligned using MUSCLE by MEGA6 [42] and then adjusted manually. MEGA6 was also used to infer phylogenetic relationships based on maximum likelihood method (ML). The ML tree was constructed by maximum likelihood method with Kimura 2-parameter model [43]. Reconstruction of ML tree are generated by the software MEGA 6, with bootstrap values set at 1000 times.

The SeqMan program was used to calculate the genetic distance between populations and count the SNP information of the *H19* gene.

The nucleotide diversity of the *H19* gene was analyzed by calculating the number of segregating sites (S), nucleotide diversity per site (π) calculated for whole sequences (π_total_), Watterson’s theta estimator (θw), and haplotype diversity (Hd) using DnaSP version 5.0 excluding insertion/deletions (indels).

To examine deviations from the neutral evolution model and reveal the history of evolution and divergence between wild boar and domesticated pigs, Tajima’s D test, Fu and Li’s D* test, Fu and Li’s F* test, Fay and Wu’s H* and HKA test were performed using DnaSP version 5.0 [44].

To observe variance in polymorphisms along the 14.6 kb *H19* gene region, including the 6 kb upstream and 6 kb downstream regions of the *H19* gene, the π and F_st_ values of *H19* gene were analyzed via sliding window with segments of 1 kb and 300 bp intervals. Besides, in order to test whether there have selection signals, the π and F_st_ of whole genome was calculated by sliding window with segments of 100 kb and 10 kb intervals by re-sequencing data. Then use the average π and F_st_ of whole genome as the sample mean to test the potential selection region using Z-test.

### High-throughput sequencing data collection

#### Re-sequencing data

To obtain additional samples for the Tibetan wild boar population and improve evolutionary analysis, re-sequencing data of Tibetan wild boars (SRA065461) were collected according to the literature published in NCBI (ftp://ftp-trace.ncbi.nlm.nih.gov/sra/sra-instant/reads/ByStudy/sra/SRP/SRP018/SRP018123/) which included 30 Tibetan wild boars, 3 wild pigs, 15 Chinese local pigs and used to analyze nucleotide diversity and F_st_ statistics along the upstream and downstream regions of the *H19* gene.

#### MeDIP data

MeDIP data of different tissues (ASA, ILB, IAD, PAD, MAD, RAD, ULB, PMM, GOM, LDM) on Tibetan wild boars (GSE30344), which including 3 female Tibetan wild boars, 3 female Rongchang pigs and 3 female Landrace, were downloaded from NCBI (ftp://ftp-trace.ncbi.nlm.nih.gov/sra/sra-instant/reads/ByStudy/sra/SRP/SRP007/SRP007462/) according to the published literature to analyze the methylation level upstream of the *H19* gene among different tissues and pig breeds.

#### Single-base resolution methylome data

Three unpublished sets of methylome data from three pig livers were used to detect the methylation pattern of the *H19* gene (the fastq file used in this article have uploaded in NCBI, the SRA accession is SRP070531).

#### RNA-seq data

Unpublished transcriptome data of three pig livers were used to detect the expression level of the *H19* gene in liver (the fastq file used in this article have uploaded in NCBI, the SRA accession is SRP070533).

### Data processing

#### Re-sequencing data analysis

Using SRA toolkit software (version 2.3.4), *.sra was transferred to *.fastq with -I --split-files parameters. Clean data were obtained by removing low quality bases, repeats, and other impurity sequences using a Perl script (Additional file 3: S9). Processed sequences were mapped to the 2.6 kb-long *H19* gene via SOAPaligner using default parameters, and SNPs were called via SOAPsnp using the –M parameter with other parameters held at their default setting. Results were used to analyze the evolution and genetic diversity of the *H19* gene by combining the sequence data of native pig and European breeds previously sequenced [45].

Clean paired-end re-sequencing data were mapped to the upstream and downstream regions of the *H19* gene, which is 56 kb-long, using Bowtie2 with default parameters (with –U parameters, other parameters all are default parameters) [46]. SNP calling was then performed using SAMtools and bcftools [47] with default parameters to obtain the SNPs of the *H19* gene in Tibetan wild boars, wild boars, and domesticated boars. Results were used to determine whether significant divergence in the *H19* gene occurred during the course of evolution and differentiation.

Clean paired-end re-sequencing data were mapped to pig genome (Sscrofa10.2) using bwa software with default parameters and called SNP using GATK software with default parameters, then based on the SNP information to calculate the average π and F_st_ of whole genome.

#### MeDIP data analysis

Similar to process the re-sequencing data, we obtain the cleaned data and then mapping the MeDIP data to the *H19* gene including the upstream and downstream of it by soapaligner using the default parameters. The number of reads was mapped to the CpG site and designated as the methylation level. Besides, in order to excluding the repeats and duplications, *Susscrofa* genome version 10.2 and *H19* was merged as the reference to be mapped by MeDIP data using soapaligner and then extract the results which mapped to *H19*.

#### Single-base resolution methylome data analysis

Low quality and repeat sequences were removed using a Perl Script to obtain clean single-base resolution methylome data. The transfer efficiency of C to T was then detected using the mitochondrial genome as reference genome by Bismark Bisulfite Mapper-v0.10.1 [48]. The clean data were mapped to the 56 kb-long *H19* gene (GenBank: AY044827) by Bismark Bisulfite Mapper-v0.10.1 using the default parameters and methylation statues of every CpG site was generated in Additional file 4: S1, Additional file 5: S2 and Additional file 6: S3 (the sam files which were produced in the process of mapping can be found in the Additional file 7: S8).

#### RNA-seq data analysis

Low-quality and repeat sequences were removed using a Perl script to obtain clean single-base resolution methylome data. Tophat software [49] was used to map the RNA-seq data to the *H19* gene (GenBank:AY044827), which was used as the reference sequence, with default parameters (the sam files which were produced in the process of mapping can be found in the Additional file 7: S8) [50]. Cufflinks software was used to remove redundancy with default parameters [51] and transcripts of the *H19* gene were then obtained, the results was shown in Additional file 8: S4, Additional file 9: S5 and Additional file 10: S6. FPKM (fragments per kilobase of exon model per million mapped reads) values were calculated as expression levels [52].

### Comparison of methylation level

Methylation levels between wild boars and domesticated pigs were analyzed using the single-base resolution liver methylome of Landrace, Enshi black pig, and a wild pig from our laboratory (unpublished). Initially, the complete *H19* gene sequence (downloaded from NCBI, AY044827.1) was indexed as the reference sequence and converted using Bismark Genome Preparation in Bismark version 0.10.1 software to allow alignment. Bowtie version2 combined with Bismark was then used to align the single base-resolution methylome sequences to the converted and indexed genome and subsequently generate the SAM format file using default parameters. Finally, methylation levels and information of each CpG site were extracted using the Bismark methylation extractor with default parameters. The methylation level of each site was calculated using the number of methylation reads of a particular site divided by the number of total reads mapped to this site. Differential methylation site between each two different pig breeds were identified by Chi-square test. Identification of Allelic specific methylation (ASM) region to identified allelic methylation status, we firstly determined SNPs with a Bayesian algorithm using SOAPsnp by aligned files which were generated by Bismark and Bowtie. All heterozygous SNPs with only two genotypes were collected and BS-seq read uniquely covering a heterozygous SNP were divided into two groups based on their assigned genotypes. We selected regions exhibiting allele-specific methylation (ASM) using the following criteria: the windows which fold change reached twice and the absolute difference methylation level reached 0.3 using five CG sites as sliding window, then overlapped the selected windows and performed *t*-test to select the allelic specific methylation (ASM) region [53]. MeDIP data [GenBank: SRP007462] from 10 tissues of Rongchang, Landrace, and Tibetan wild boars published in NCBI were downloaded. After cleaning, SOAPaligner and SOAPsnp were used to align the data to the complete *H19* reference gene sequence and extract the mapping reads of every site. The number of reads was defined with respect to the methylation level of each site. In order to identify the difference of methylation analysis, ANOVA or pairwise comparisons using *t* tests was employed for comparisons of more than two groups, Student’s *t*-test was used for two group comparisons. Resulting *P* values of pairwise comparisons using *t* tests were corrected with adjusted Bonferroni method.

### Ethics

Animals care and all the experimentation in this study were carried out in accordance with the pre-approved guidelines from Regulation Proclamation No.5 of the Standing Committee of Hubei People’s Congress. All experimental procedures and sample collection methods were approved by the Institutional Animal Care and Use Committee of Huazhong Agricultural University, Wuhan, China (permit HZAUSW2015-0003). We obtained a written informed consent from the animal owners to take blood and tissue samples.

### Consent to publish

Not applicable.

### Availability of data and materials

Three unpublished sets of methylome data from three pig livers were used to detect the methylation pattern of the *H19* gene (the fastq file used in this article have uploaded in NCBI, the SRA accession is SRP070531). Unpublished transcriptome data of three pig livers were used to detect the expression level of the *H19* gene in liver (the fastq file used in this article have uploaded in NCBI, the SRA accession is SRP070533).

## Additional files

Additional file 1: Table S1-S5 and Figure S1-S5. (PDF 880 kb) Additional file 2: S7. The information of SNP and ASM sites in ES, LB, GZWB. (XLSX 30 kb) Additional file 3: S9. Perl scripts which used in this article, including clean script, perl scripts which were used to calculate pi and Fst with sliding window, and perl scripts which were used to analyze ASM. (RAR 35 kb) Additional file 4: S1. The result of CpG report of *H19* completely sequence (GenBank: AY044827) in Enshi black pig according to the single base methylation data which has not published. (TXT 198 kb) Additional file 5: S2. The result of CpG report of *H19* completely sequence (GenBank: AY044827) in Guizhou wild boar according to the single base methylation data which has not published. (TXT 197 kb) Additional file 6: S3. The result of CpG report of *H19* completely sequence (GenBank: AY044827) in Landrace according to the single base methylation data which has not published. (TXT 197 kb) Additional file 7: S8. The mapping file of unpublished single-base resolution methylation data and transcriptome of liver in Enshi pig, Landrace and Wild pig (sam file). (RAR 16698 kb) Additional file 8: S4. The transcript information of *H19* completely sequence (GenBank: AY044827) in Enshi black pig according to the transcriptome data. (TXT 4 kb) Additional file 9: S5. The transcript information of *H19* completely sequence (GenBank: AY044827) in Guizhou wild boar according to the transcriptome data. (TXT 1 kb) Additional file 10: S6. The transcript information of *H19* completely sequence (GenBank: AY044827) in Landrace according to the transcriptome data. (TXT 4 kb)

## Abbreviations

ASA: abdominal subcutaneous adipose
ASM: allele specific methylation
BWS: Beckwith-Wiedemann syndrome
CC: Central China
DMRs: differential methylated regions
DMS: differential methylation sites
EU: European
F_st_: F statistics
GOM: greateromentum
Hd: haplotype diversity
ILB: inner lipid of back
indels: insertion/deletions
JH: Jianghai
LDM: longissimus dorsi muscle
lncRNA: long non-coding RNA
MAD: mesentery adipose
MeDIP: methylated DNA Immunoprecipitation
MeDIP-seq: methylated DNA immunoprecipitation sequencing
NC: North China
PCR: polymerase chain reaction
PMM: psoas major muscle
RAD: retroperitoneal adipose
SC: South China
SNPs: single nucleotide polymorphisms
SRS: Silver-Russell Syndrome
SW: Southwest China
TB: Tibetan wild boar
ULB: upper lipid of back
WB.S: wild boar of Sichuan in China
θw: Watterson’s theta estimator
π: nucleotide diversity

## References

[1] Larson G, Dobney K, Albarella U, Fang M, Matisoo-Smith E, Robins J, Lowden S, Finlayson H, Brand T, Willerslev E (2005). Worldwide phylogeography of wild boar reveals multiple centers of pig domestication. Science.

[2] Ai H, Fang X, Yang B, Huang Z, Chen H, Mao L, Zhang F, Zhang L, Cui L, He W (2015). Adaptation and possible ancient interspecies introgression in pigs identified by whole-genome sequencing. Nat Genet.

[3] SCHERF BD. World watch list for domestic animal diversity, 3rd edn; 2000.

[4] Fang M, Andersson L (2006). Mitochondrial diversity in European and Chinese pigs is consistent with population expansions that occurred prior to domestication. Proc Biol Sci.

[5] Sang Y, Bergkamp J, Blecha F (2014). Molecular evolution of the porcine type I interferon family: subtype-specific expression and antiviral activity. PLoS One.

[6] Albert FW, Somel M, Carneiro M, Aximu-Petri A, Halbwax M, Thalmann O, Blanco-Aguiar JA, Plyusnina IZ, Trut L, Villafuerte R (2012). A comparison of brain gene expression levels in domesticated and wild animals. PLoS Genet.

[7] Bergman IM, Rosengren JK, Edman K, Edfors I (2010). European wild boars and domestic pigs display different polymorphic patterns in the Toll-like receptor (TLR) 1, TLR2, and TLR6 genes. Immunogenetics.

[8] Esteve A, Ojeda A, Huang LS, Folch JM, Perez-Enciso M (2011). Nucleotide variability of the porcine SERPINA6 gene and the origin of a putative causal mutation associated with meat quality. Anim Genet.

[9] Zhou ZY, Li AM, Adeola AC, Liu YH, Irwin DM, Xie HB, Zhang YP (2014). Genome-wide identification of long intergenic noncoding RNA genes and their potential association with domestication in pigs. Genome Biol Evol.

[10] Wang KC, Yang YW, Liu B, Sanyal A, Corces-Zimmerman R, Chen Y, Lajoie BR, Protacio A, Flynn RA, Gupta RA (2011). A long noncoding RNA maintains active chromatin to coordinate homeotic gene expression. Nature.

[11] Jiang H, Yu Y, Xun P, Zhang J, Luo G, Wang Q (2014). Maternal mRNA expression levels of H19 are inversely associated with risk of macrosomia. Arch Med Sci.

[12] Le F, Wang LY, Wang N, Li L, le Li J, Zheng YM, Lou HY, Liu XZ, Xu XR, Sheng JZ (2013). In vitro fertilization alters growth and expression of Igf2/H19 and their epigenetic mechanisms in the liver and skeletal muscle of newborn and elder mice. Biol Reprod.

[13] Borensztein M, Monnier P, Court F, Louault Y, Ripoche MA, Tiret L, Yao Z, Tapscott SJ, Forne T, Montarras D (2013). Myod and H19-Igf2 locus interactions are required for diaphragm formation in the mouse. Development.

[14] Dey BK, Pfeifer K, Dutta A (2014). The H19 long noncoding RNA gives rise to microRNAs miR-675-3p and miR-675-5p to promote skeletal muscle differentiation and regeneration. Genes Dev.

[15] Vercelli D (2004). Genetics, epigenetics, and the environment: switching, buffering, releasing. J Allergy Clin Immunol.

[16] Wagner JR, Busche S, Ge B, Kwan T, Pastinen T, Blanchette M (2014). The relationship between DNA methylation, genetic and expression inter-individual variation in untransformed human fibroblasts. Genome Biol.

[17] Li H, Yu B, Li J, Su L, Yan M, Zhu Z, Liu B (2014). Overexpression of lncRNA H19 enhances carcinogenesis and metastasis of gastric cancer. Oncotarget.

[18] Luo M, Li Z, Wang W, Zeng Y, Liu Z, Qiu J (2013). Upregulated H19 contributes to bladder cancer cell proliferation by regulating ID2 expression. FEBS J.

[19] Abi Habib W, Azzi S, Brioude F, Steunou V, Thibaud N, Das Neves C, Le Jule M, Chantot-Bastaraud S, Keren B, Lyonnet S (2014). Extensive investigation of the IGF2/H19 imprinting control region reveals novel OCT4/SOX2 binding site defects associated with specific methylation patterns in Beckwith-Wiedemann syndrome. Hum Mol Genet.

[20] Gucev ZS, Saranac L, Jancevska A, Tasic V (2013). The degree of H19 hypomethylation in children with Silver-Russel syndrome (SRS) is not associated with the severity of phenotype and the clinical severity score (CSS). Prilozi.

[21] Pidsley R, Dempster E, Troakes C, Al-Sarraj S, Mill J (2012). Epigenetic and genetic variation at the IGF2/H19 imprinting control region on 11p15.5 is associated with cerebellum weight. Epigenetics.

[22] Hubisz MJ, Pollard KS, Siepel A (2011). PHAST and RPHAST: phylogenetic analysis with space/time models. Brief Bioinform.

[23] Kumar S, Tamura K, Nei M (1994). MEGA: Molecular Evolutionary Genetics Analysis software for microcomputers. Comput Appl Biosci.

[24] Li M, Tian S, Jin L, Zhou G, Li Y, Zhang Y, Wang T, Yeung CK, Chen L, Ma J (2013). Genomic analyses identify distinct patterns of selection in domesticated pigs and Tibetan wild boars. Nat Genet.

[25] Fang M, Larson G, Ribeiro HS, Li N, Andersson L (2009). Contrasting mode of evolution at a coat color locus in wild and domestic pigs. PLoS Genet.

[26] McKenna A, Hanna M, Banks E, Sivachenko A, Cibulskis K, Kernytsky A, Garimella K, Altshuler D, Gabriel S, Daly M (2010). The Genome Analysis Toolkit: a MapReduce framework for analyzing next-generation DNA sequencing data. Genome Res.

[27] Schlotterer C (2003). Hitchhiking mapping--functional genomics from the population genetics perspective. Trends Genet.

[28] Yu HS, Shen YH, Yuan GX, Hu YG, Xu HE, Xiang ZH, Zhang Z (2011). Evidence of selection at melanin synthesis pathway loci during silkworm domestication. Mol Biol Evol.

[29] Tajima F (1989). Statistical method for testing the neutral mutation hypothesis by DNA polymorphism. Genetics.

[30] Fu YX, Li WH (1993). Statistical tests of neutrality of mutations. Genetics.

[31] Fay JC, Wu CI (2000). Hitchhiking under positive Darwinian selection. Genetics.

[32] Venkatraman A, He XC, Thorvaldsen JL, Sugimura R, Perry JM, Tao F, Zhao M, Christenson MK, Sanchez R, Yu JY (2013). Maternal imprinting at the H19-Igf2 locus maintains adult haematopoietic stem cell quiescence. Nature.

[33] Wu J, Xiang H, Qi Y, Yang D, Wang X, Sun H, Wang F, Liu B (2014). Adaptive evolution of the STRA6 genes in mammalian. PLoS One.

[34] Alders M, Bliek J, vd Lip K, vd Bogaard R, Mannens M (2009). Determination of KCNQ1OT1 and H19 methylation levels in BWS and SRS patients using methylation-sensitive high-resolution melting analysis. Eur J Hum Genet.

[35] Bouwland-Both MI, van Mil NH, Stolk L, Eilers PH, Verbiest MM, Heijmans BT, Tiemeier H, Hofman A, Steegers EA, Jaddoe VW (2013). DNA methylation of IGF2DMR and H19 is associated with fetal and infant growth: the generation R study. PLoS One.

[36] Gao T, He B, Pan Y, Gu L, Chen L, Nie Z, Xu Y, Li R, Wang S (2014). H19 DMR methylation correlates to the progression of esophageal squamous cell carcinoma through IGF2 imprinting pathway. Clin Transl Oncol.

[37] Murrell A, Ito Y, Verde G, Huddleston J, Woodfine K, Silengo MC, Spreafico F, Perotti D, De Crescenzo A, Sparago A (2008). Distinct methylation changes at the IGF2-H19 locus in congenital growth disorders and cancer. PLoS One.

[38] Wang D, Song Y, Huang Y, Duan F, Lv Q, Ouyang H, Lai L, Li Z (2014). Genomic imprinting analysis of Igf2/H19 in porcine cloned fetuses using parthenogenetic somatic cells as nuclear donors. Biotechnol Lett.

[39] Schultz MD, He Y, Whitaker JW, Hariharan M, Mukamel EA, Leung D, Rajagopal N, Nery JR, Urich MA, Chen H (2015). Human body epigenome maps reveal noncanonical DNA methylation variation. Nature.

[40] Jiang L, Mu J, Zhang Q, Ni T, Srinivasan P, Rayavara K, Yang W, Turner L, Lavstsen T, Theander TG (2013). PfSETvs methylation of histone H3K36 represses virulence genes in Plasmodium falciparum. Nature.

[41] Barlow DP, Bartolomei MS. Genomic imprinting in mammals. Cold Spring Harb Perspect Biol. 2014, 6(2). pii: a018382. 10.1101/cshperspect.a018382 PMC3941233 24492710

[42] Tamura K, Stecher G, Peterson D, Filipski A, Kumar S (2013). MEGA6: Molecular Evolutionary Genetics Analysis version 6.0.. Mol Biol Evol.

[43] Kimura M (1980). A simple method for estimating evolutionary rates of base substitutions through comparative studies of nucleotide sequences. J Mol Evol.

[44] Librado P, Rozas J (2009). DnaSP v5: a software for comprehensive analysis of DNA polymorphism data. Bioinformatics.

[45] Li R, Yu C, Li Y, Lam TW, Yiu SM, Kristiansen K, Wang J (2009). SOAP2: an improved ultrafast tool for short read alignment. Bioinformatics.

[46] Langmead B, Trapnell C, Pop M, Salzberg SL (2009). Ultrafast and memory-efficient alignment of short DNA sequences to the human genome. Genome Biol.

[47] Ramirez-Gonzalez RH, Bonnal R, Caccamo M, Maclean D (2012). Bio-samtools: Ruby bindings for SAMtools, a library for accessing BAM files containing high-throughput sequence alignments. Source Code Biol Med.

[48] Krueger F, Andrews SR (2011). Bismark: a flexible aligner and methylation caller for Bisulfite-Seq applications. Bioinformatics.

[49] Trapnell C, Pachter L, Salzberg SL (2009). TopHat: discovering splice junctions with RNA-Seq. Bioinformatics.

[50] Kim D, Salzberg SL (2011). TopHat-Fusion: an algorithm for discovery of novel fusion transcripts. Genome Biol.

[51] Amoore JN (1993). Assessment of oscillometric non-invasive blood pressure monitors using the Dynatech Nevada CuffLink analyser. J Med Eng Technol.

[52] Mortazavi A, Williams BA, McCue K, Schaeffer L, Wold B (2008). Mapping and quantifying mammalian transcriptomes by RNA-Seq. Nat Methods.

[53] Bonasio R, Li Q, Lian J, Mutti NS, Jin L, Zhao H, Zhang P, Wen P, Xiang H, Ding Y (2012). Genome-wide and caste-specific DNA methylomes of the ants Camponotus floridanus and Harpegnathos saltator. Curr Biol.

[54] Halley P, Kadakkuzha BM, Faghihi MA, Magistri M, Zeier Z, Khorkova O, Coito C, Hsiao J, Lawrence M, Wahlestedt C (2014). Regulation of the apolipoprotein gene cluster by a long noncoding RNA. Cell Rep.

[55] Felsenstein J (1985). Confidence limits on phylogenies: an approach using the bootstrap. Evolution.

