# Supplementary material for: Molecular microevolution and epigenetic patterns of the long non-coding gene H19 show its potential function in pig domestication and breed divergence
Source: BMC Evol Biol. 2016 Apr 23;16:87. doi: 10.1186/s12862-016-0657-5 (PMC4841954; doi:10.1186/s12862-016-0657-5)
Supplement: Additional file 1: — Table S1-S5 and Figure S1-S5. (PDF 880 kb) [file 12862_2016_657_MOESM1_ESM.pdf]

Supplementary information

Table S1.. Summery of Primers

	Primer_F	Primer_R	length	Anealing Tm
Sus_H19_1	TATGCGGACGATGCCAGAC	GCCACGACCTGAAACCAAAA	602	60
Sus_H19_2	TAGGCAGGGCAACATCGG	TGGTCTCGTCAGTGCCCTTG	912	63
Sus_H19_3	CAAGGGCACTGACGAGACCA	TCCCTCCAAGCACATCCTG	345	60
Sus_H19_4	GGACGCCAGGATGTGCTTG	TGAGGTCTGCTCGGTGGTC	1034	63
Sus_H19_5	TCCTTTGCTCAGAAGACTCGG	TGTGCTTTGTGGGGTAGGG	654	61

Table S2.. SNP information of different population

Location	Sample ID	n	1664bp	1818bp	1993bp	2020bp	2028bp	2050bp	2292bp	2456bp
NC	Hetaodaer	10	C	G	C	C/T$_{(9/1)}$	G/A$_{(7/3)}$	C/T$_{(7/3)}$	C	C
	Hanjiang	13	C	G	C	C/T$_{(11/2)}$	G	C/T$_{(12/1)}$	C/T$_{(11/2)}$	C
CC	Ningxiang	23	C	G/A$_{(15/8)}$	C/T$_{(18/5)}$	C/T$_{(18/5)}$	G	C	C/T$_{(17/6)}$	C
	Tongcheng	19	C	G/A$_{(16/3)}$	C/T$_{(4/15)}$	C	G	C/T$_{(4/15)}$	C	C
JH	Jiaxing	8	C	G	C	C	G	C	C	C/T$_{(5/3)}$
	Jiangquhai	21	C	G	C	C/T$_{(13/8)}$	G	C	C/T$_{(10/11)}$	C
SC	Luchuan	18	C	G/A$_{(1/17)}$	C	C	G	C	C	C/T$_{(14/4)}$
	Longlin	8	C	G/A$_{(7/1)}$	C	C	G/A$_{(7/1)}$	C/T$_{(6/2)}$	C/T$_{(5/3)}$	C
	Huaizhu	18	C	G/A$_{(9/9)}$	C/T$_{(12/6)}$	C	G	C/T$_{(6/12)}$	C	C/T$_{(16/2)}$
	Diannan	12	C/T$_{(8/4)}$	G/A$_{(10/2)}$	C/T$_{(11/1)}$	C	G/A$_{(9/3)}$	C/T$_{(4/8)}$	C	C
	Hainan	19	C	G	C/T$_{(11/8)}$	C	G/A$_{(17/2)}$	C/T$_{(9/10)}$	C	C
	Dongshan	9	C	G/A$_{(4/5)}$	C	C	G	C/T$_{(2/7)}$	C	C
SW	Wujin	17	C	G	C	C	G	C	C	C
	Neijiang	6	C	G	C	C	G	C/T$_{(4/2)}$	C/T$_{(2/4)}$	C
EU	Duroc	8	C	G/A$_{(7/1)}$	C	C	G	C/T$_{(5/3)}$	C	C
	Landrace	5	C	G	C/T$_{(1/4)}$	C	G	C/T$_{(1/4)}$	C	C
	Yorkshire	20	C	G	C	C	G	C	C	C
TB	Tibetan wild boar	27	C	G	C	C/T$_{(26/1)}$	G	C	C	C
WB.S	Sichuan wild boar	3	C	G	C	C/T$_{(1/2)}$	G	C	C	C

NC: North China; CC: Central China; JH: Jianghai; SC: South China; SW: Southwest China; EU: European; TB: Tibetan; WB.S: Wild boar of southwest china; n: the number of samples.

Table S3.. Summary of SNP and gene frequency of H19

SNP name	SNP position	SNP position from first exon	genotype	NC	CC	JH	SC	SW	EU	WB.S	TB
AY044827:g.36061C>T	36061	1664	CC	23	42	29	80	23	33	3	27
			TT	0	0	0	4	0	0	0	0
			C frequency	100.00%	100.00%	100.00%	95.24%	100.00%	100.00%	100.00%	100.00%
			T frequency	0.00%	0.00%	0.00%	4.76%	0.00%	0.00%	0.00%	0.00%
AY044827:g.36215G>A	36215	1818	GG	23	31	29	50	23	32	3	27
			GA	0	2	0	8	0	0	0	0
			AA	0	9	0	26	0	1	0	0
			G frequency	100.00%	76.19%	100.00%	64.29%	100.00%	96.97%	100.00%	100.00%
			A frequency	0.00%	23.81%	0.00%	35.71%	0.00%	3.03%	0.00%	0.00%
AY044827:g.36390C>T	36390	1993	CC	23	22	29	69	23	29	3	27
			CT	0	5	0	8	0	1	0	0
			TT	0	15	0	7	0	3	0	0
			C frequency	100.00%	58.33%	100.00%	86.90%	100.00%	89.39%	100.00%	100.00%
			T frequency	0.00%	41.67%	0.00%	13.10%	0.00%	10.61%	0.00%	0.00%
AY044827:g.36417C>T	36417	2020	CC	20	37	29	84	23	33	1	26
			CT	0	3	0	0	0	0	2	0
			TT	3	2	0	0	0	0	0	1
			C frequency	86.96%	91.67%	100.00%	100.00%	100.00%	100.00%	50.00%	96.30%
			T frequency	13.04%	8.33%	0.00%	0.00%	0.00%	0.00%	50.00%	3.70%
AY044827:g.36425G>A	36425	2028	GG	20	42	29	78	23	33	3	27
			GA	0	0	0	1	0	0	0	0
			AA	3	0	0	5	0	0	0	0
			G frequency	86.96%	100.00%	100.00%	93.45%	100.00%	100.00%	100.00%	100.00%
			A frequency	13.04%	0.00%	0.00%	6.55%	0.00%	0.00%	0.00%	0.00%
AY044827:g.36447C>T	36447	2050	CC	17	16	21	46	21	26	3	26
			CT	1	10	0	9	0	0	0	0
			TT	5	16	8	29	2	7	0	1
			C frequency	76.09%	50.00%	72.41%	60.12%	91.30%	78.79%	100.00%	96.30%
			T frequency	23.91%	50.00%	27.59%	39.88%	8.70%	21.21%	0.00%	3.70%
AY044827:g.36689C>T	36689	2292	CC	21	36	18	81	20	33	3	27
			CT	0	4	0	0	0	0	0	0
			TT	2	2	11	3	3	0	0	0
			C frequency	91.30%	90.48%	62.07%	96.43%	86.96%	100.00%	100.00%	100.00%
			T frequency	8.70%	9.52%	37.93%	3.57%	13.04%	0.00%	0.00%	0.00%
AY044827:g.36853C>T	36853	2456	CC	23	42	26	78	23	33	3	27
			CT	0	0	0	0	0	0	0	0
			TT	0	0	3	6	0	0	0	0
			C frequency	100.00%	100.00%	89.66%	92.86%	100.00%	100.00%	100.00%	100.00%
			T frequency	0.00%	0.00%	10.34%	7.14%	0.00%	0.00%	0.00%	0.00%

Table S4.. Summary statistics of sequence diversity

Species	n	S	π	θ	θ w	Hd	Tajima's D	D*	F*	H*
NC	23	5	0.00270	0.00271	0.00271	0.87400	-0.01413	0.15681	0.12420	1.22530
SC	84	7	0.00203	0.00202	0.00202	0.80900	0.001575	0.49494	0.39442	1.17468
CC	42	6	0.00127	0.00203	0.00203	0.52100	-1.02463	0.50103	0.02625	0.91521
SW	27	5	0.00194	0.00217	0.00217	0.66500	-0.31771	0.56193	0.34408	1.16129
JH	29	3	0.00205	0.00255	0.00223	0.76100	-0.24153	-1.48809	-1.29876	0.69951
TB	27	4	0.00071	0.00130	0.00130	0.39900	-1.20640	0.07714	-0.33926	
WB.S	3	1	0.00063	0.00068	0.00068	0.50000	-0.61237	-0.61237	-0.47871	0.33333
EU	33	3	0.00264	0.00277	0.00246	0.87500	0.21198	0.02128	0.09344	1.09470

***P<0.001;**P<0.02;*P<0.05; n: sample size; S: the number of segregating sites; π : Nucleotide diversity; θ or θw: Theta (per site) from Eta or S, the Watterson estimator of 4Nμ， Eta (η) is the total number of mutations, and S is the number of segregating (polymorphic) sites; Hd: Haplotype diversity; D*: Fu and Li's D* test; F*: Fu and Li's F* test; H*: Fay and Wu's H* test.

Table S5.. The information of differential methylation sites

ES and GZWB			ES and Landrace			GZWB and Landrace		
region	Pos(+/-)	P-value	region	Pos(+/-)	P-value	region	Pos(+/-)	P-value
upstream	32472	0.034487	upstream	32530	1.75E-07	upstream	32530	0.002623
upstream	32478	0.015877	upstream	33250	0.025285	upstream	32826	0.036236
upstream	33694	9.36E-06	upstream	33525	0.008365	upstream	33648	0.002246
gene	35577	0.014017	upstream	33539	0.019602	upstream	33684	0.002087
gene	36410	0.024201	upstream	33545	0.012841	upstream	33694	0.006581
downstream	37493	0.020902	upstream	33648	0.009388	upstream	34183	0.000310
			upstream	33663	0.000298	gene	34818	0.001677
			upstream	33684	0.000556	gene	35438	0.004100
			upstream	33783	0.047478	downstream	37827	0.000440
			gene	35052	0.021636			
			gene	35070	0.024889			
			gene	35156	0.039924			

All sites are differential methylation sties (DMSs). The site was marked by red is located in the allele specific methylation (ASM) region. Because DMSs were calculated in every CpG site, the position was written in Pos(+/-).

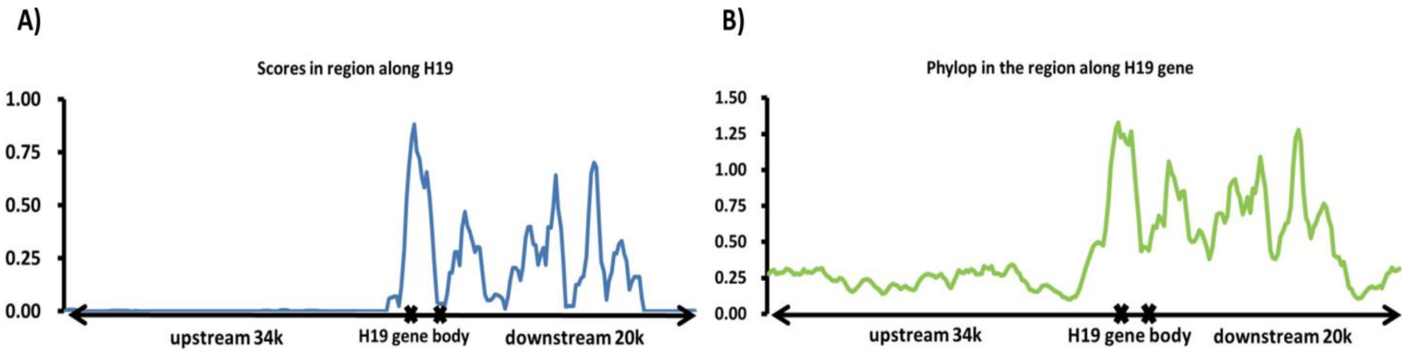

Figure S1.. PhastCon score and phyloP value of within flanking region of H19 among 18 species. The phastCons score (A) and the phylop value (B) within flanking regions, which was extended to the 34 kb upstream and 20 kb downstream, of H19 among 18 species was calculated using the Phast software and then was calculated using sliding window for segments of 1000 bp with 300 bp intervals.

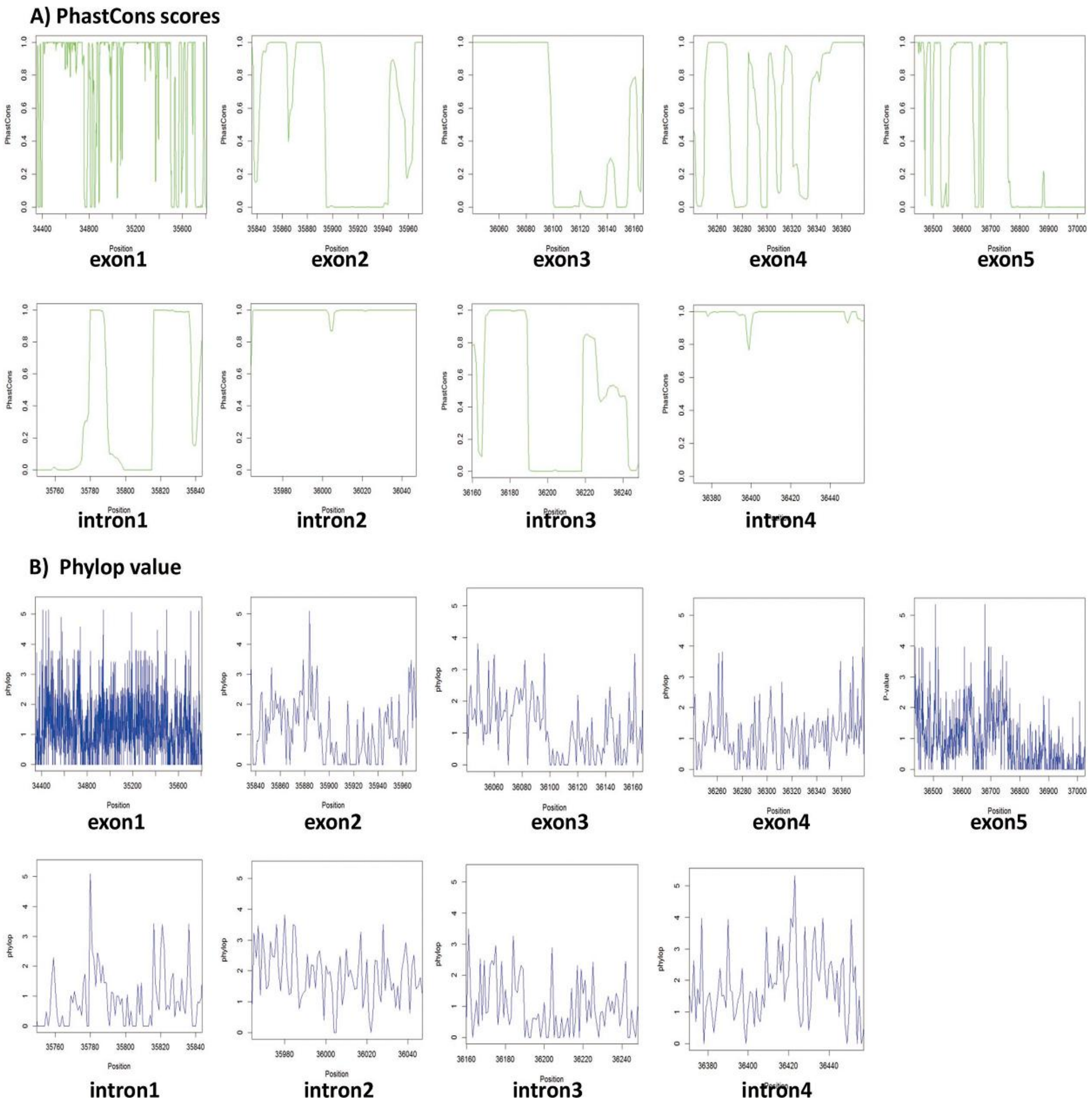

Figure S2.. PhastCon (A) and phyloP (B) of H19. The phastCon value and phyloP value of 5 exons (above) and introns (below) was caulaed by Phast software using H19 homologous sequences of 18 species.

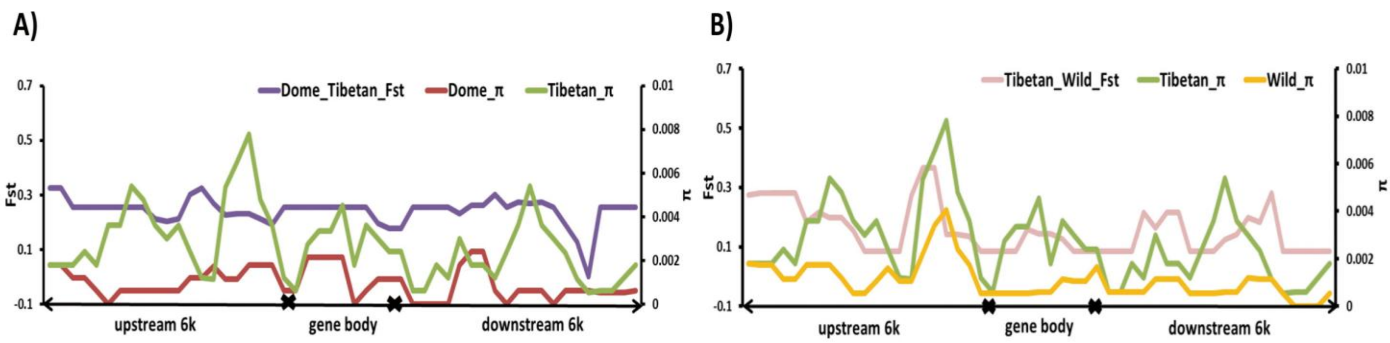

Figure S3.. Patterns of nucleotide diversity and pair-wise population differentiation of H19 locus between domesticated pigs (Dome) and Tibetan, also between Tibetan and wild boars (Wild). The cleaned Tibetan re-sequencing data was aligned to the H19 completely sequence which contains 34 kb upstream region, H19 gene and downstream 20 kb region using bowtie2, then SNP was calling using samtools software and the π value and Fst was calculated using sliding window analysis between Tibetan and domesticated pigs (A) , also between Tibetan and wild pigs (B).

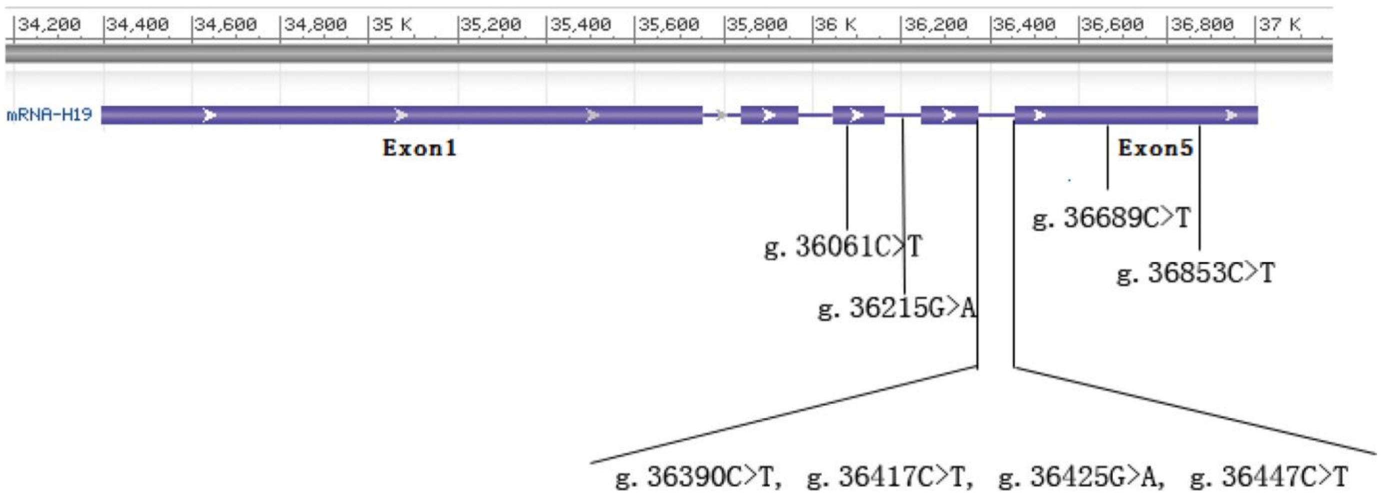

Figure S4.. The location of SNP in H19. The location of 8 SNP that we identified in different domestication pig breeds and Asian wild pigs in the region of 1660 to 2460 of H19 gene.

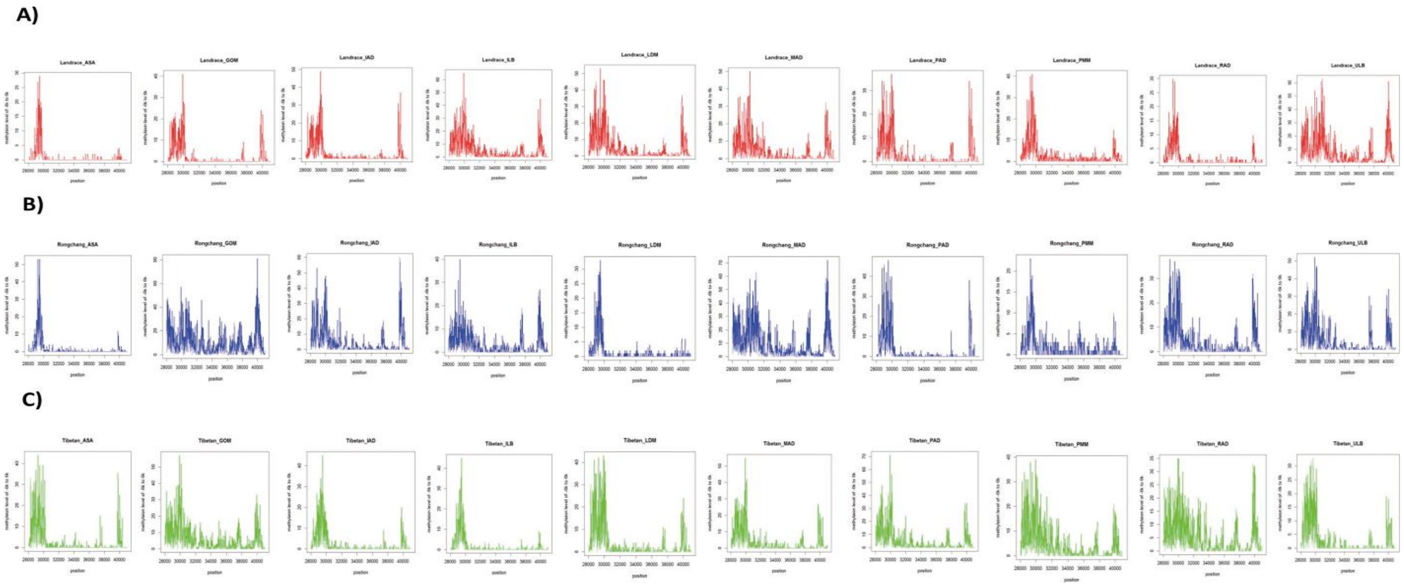

Figure S5.. Methylation level of H19, the upstream 6 kb and downstream 6 kb of 10 tissues of Landrace (A), Rongchang pig (B) and Tibetan pig (C).
